# A prospective study to evaluate high dose daptomycin pharmacokinetics and pharmacodynamics in *Staphylococcus* spp. infective endocarditis

**DOI:** 10.1177/20499361241296232

**Published:** 2025-01-08

**Authors:** Simona De Gregori, Annalisa De Silvestri, Mara Capone, Vincenzina Monzillo, Paola Giordani, Raffaele Bruno, Elena Seminari

**Affiliations:** Clinical and Experimental Pharmacokinetics Unit, Department of Diagnostic Medicine, Fondazione IRCCS Policlinico San Matteo, Pavia, Italy; SSD Biostatistica e Clinical Trial Center -Direzione Scientifica, Fondazione IRCCS Policlinico San Matteo, Pavia, Italy; Clinical and Experimental Pharmacokinetics Unit, Department of Diagnostic Medicine, Fondazione IRCCS Policlinico San Matteo, Pavia, Italy; Microbiology and Virology Unit, Fondazione IRCCS Policlinico San Matteo, Pavia, Italy; Clinica di Malattie Infettive, Fondazione IRCCS Policlinico San Matteo, Pavia, Italy; Department of Medical, Surgical, Diagnostic and Pediatric Science, University of Pavia, Pavia, Italy; Clinica di Malattie Infettive, Fondazione IRCCS Policlinico San Matteo, Pavia, Italy; Department of Medical, Surgical, Diagnostic and Pediatric Science, University of Pavia, Pavia, Italy; Clinica di Malattie Infettive, Fondazione IRCCS Policlinico San Matteo, Pavia 27100, Italy

**Keywords:** AUC, daptomycin, infectious endocarditis, pharmacokinetics, PK/PD, *Staphylococcus aureus*, *Staphylococcus epidermidis*

## Abstract

**Background:**

Daptomycin pharmacokinetics and pharmacodynamics data relative to higher doses in patients are necessary for clinical practice.

**Objectives:**

A monocentric, prospective study that enrolled patients with a diagnosis of *Staphylococcus* spp. infective endocarditis treated with daptomycin according to clinical practice, to evaluate the pharmacokinetics/pharmacodynamics of different daptomycin daily doses (group A: 8–10 and group B: 11–12 mg/kg).

**Design and methods:**

A monocentric, prospective, cohort study that enrolled patients with a diagnosis of *Staphylococcus* spp. infective endocarditis treated with daptomycin. Daptomycin was administered by intravenous infusion over a 30-min period for at least five consecutive days before PK study.

**Results:**

Twenty-two patients were included. Native valve infectious endocarditis (IE) was diagnosed in 9 patients, prosthetic valve IE was diagnosed in 10 patients and 3 patients had concomitant intracardiac device infections. All patients showed a microbiologic response with negative blood cultures by day 5 (1–3 interquartile rate (IQR) 3–8). The median calculated AUC_0–24_ was 1298 (1–3 IQR 1069–1484) and 1459 (1–3 IQR 1218–1711) µg*h/mL, with the corresponding clearance of 0.49 (1–3 IQR 0.37–0.57) and 0.57 (1–3 IQR 0.40–0.71) L/h, respectively. A value of area under the curve/minimum inhibitory concentration (AUC/MIC) > 666 was reached by all patients; however, 4 out of 15 patients in group A and 1 out of 14 patients in group B did not reach the pharmacokinetic/pharmacodynamic (PK/PD) target of 1061; therefore, AUC/MIC equal to or above 1061 was reached by 73.3% in group A and 92.9% in group B.

**Conclusion:**

From a PK/PD point of view, all patients reached the value of AUC/MIC > 666, while roughly 70% of patients in group A and 90% in group B reached the target value of AUC/MIC>1061. Even if this cut-off value is arbitrary, 11–12 mg/kg daily dose could be taken into consideration in case of serious infections characterised by a high inoculum or in cases of prosthetic valve infections.

## Introduction

The incidence of *Staphylococcus* spp. endocarditis has recently increased, comprising up to 40% of all endocarditis cases,^1^ with in-hospital mortality that can reach 20%.^2^ Daptomycin is a bactericidal drug that acts by binding to the Gram-positive bacterial cell membrane and is proven to be effective in the treatment of *Staphylococcus* endocarditis,^3^ mostly in combination with β-lactams in the case of methicillin-resistant strains.^4^ Daptomycin is approved for complicated skin and soft-tissue infections, right-sided infective endocarditis due to *Staphylococcus aureus* and *S. aureus* bacteraemia, at the daily dose of 4 mg/kg for soft-tissue infection and 6 mg/kg for endocarditis and bacteraemia.^5^ Therapeutic failures with the emergence of resistance have been reported in the case of left-sided endocarditis, particularly in patients treated with standard dose.^6^ Most authors therefore suggest that daily doses of 8–10 mg/kg should be used to treat serious infections.^7–9^ The potential clinical response of higher doses (8–12 mg/kg) is supported by *in vitro* pharmacokinetic/pharmacodynamic models utilizing *S. aureus* high inocula.^10,11^ As a result, there are published studies with high-dose daptomycin, treatment may refer to widely varying daily doses ranging from 6.1 mg/kg to >12 mg/kg.^7–9^ Current European Society of Cardiology (ESC) guidelines suggest using daptomycin at a dose of 10 mg/kg daily,^12^ higher doses such as 10–12 mg/kg have been variously utilized on the basis of infection severity.^7,8,13^

The area under the curve/minimum inhibitory concentration (AUC/MIC) ratio is the PK/PD index that, among other variables (i.e., source control, resistance pattern) best correlates with daptomycin therapeutic success; AUC/MIC value associated clinical response varies between >666 and ⩾1061^14^ (with strains exhibiting a daptomycin MIC of 0.5 µg/mL).

The objectives of the present study were to compare pharmacokinetic and pharmacodynamic parameters relative to two different daptomycin dosages (8–10 and 11–12 mg/kg) in patients with *Staphylococcus* endocarditis and the incidence of microbiological failures during treatment in the two groups.

## Patients and methods

### Patients

Adult patients with a diagnosis of *Staphylococcus* spp. infective endocarditis diagnosed according to the Modified Duke criteria,^15^ hospitalised in the Infectious Diseases Unit of the Fondazione IRCCS Policlinico San Matteo of Pavia from 1st January 2020 until 31st March 2023, were included in the present study and treated with daptomycin. Exclusion criteria were mainly represented by concomitant kidney failure (creatinine clearance < 40 mL/min) and pregnancy. Patients on concomitant methadone treatment have been excluded from the present analysis as their plasma concentration has been demonstrated to be lower than expected, as previously described.^16^

The monocentric, prospective, cohort study was approved by the local ethics committee. The objective of the study was to evaluate the pharmacokinetic/pharmacodynamic parameters of different daily doses of daptomycin (8–10 and 11–12 mg/kg) in an intra-patient and inter-patient design (Daptomycin PK/PD study, protocol number 20200006979). All participants signed an informed consent form before the initiation of treatment and data/biological samples collection. According to the study design, serum samples to ascertain the pharmacokinetics features of daptomycin should have been collected at days 5 and 10 of treatment, with patients receiving two different daptomycin doses 5 days apart plus the anti-staphylococcal β-lactam cefazolin (2 g every 8 h). Due to the COVID-19 pandemic, we could not completely fulfil the study design, and patients were treated with either 8–10 mg/kg (group A) or 11–12 mg/kg (group B), as per routine clinical treatment. In six patients, PK data relative to both doses are available, as per the initial protocol. If the patient had a methicillin-resistant *Staphylococcus* IE cefazolin 2 g every 8 h was added. Surveillance blood cultures were performed every 48 h, to ascertain the clearance of S*taphylococcus* spp from the blood. Microbiological failure was defined by persistent or breakthrough infection during IE treatment. Serum creatinine kinase (CK) was evaluated weekly for safety reasons as well as sign and symptoms of daptomycin-induced eosinophilic pneumonia.

#### Statistical analysis

The sample size was based on feasibility; with 15 curves per group, it was possible to achieve an 80% power to find a significant effect (alpha error of 5%) with an effect size of 1.05 (i.e., the standard deviation is 5% greater than mean difference). Data are reported as median and interquartile rate (IQR).

Dose group, gender, age and creatinine levels were included as covariates using original data in a fixed part of multilevel (patients and curve) linear mixed models using patients as random factors to evaluate their possible effect on daptomycin pharmacokinetics. Multilevel modelling is designed to explore and analyse data that come from populations. In any complex structure, we can identify atomic units (in this case curves) and higher level unit (in this case patients). Treating patients as a random effect will correct standard errors and furnish an estimate of between-group variance. Introducing some covariate in a fixed part of the models helps to explain and so reduce unexplained between-group variance. Results are expressed as beta coefficient with their 95% confidence interval (95% CI) and presented with term-specific *p* values; the beta coefficient represents the mean variation of outcomes for unit change of quantitative predictors or between levels of categorical or ordinal predictors.

##### Blood collection

Daptomycin was administered by intravenous infusion over a 30-min period for at least 5 consecutive days before the PK study. Blood samples were collected by venepuncture into potassium ethylenediaminetetraacetic acid (EDTA)-containing tubes, immediately before the daptomycin IV administration (*C*_trough_) and 0.5, 1, 2, 4, 12 and 24 h after starting infusion. Tubes were centrifuged at 10,400 rpm for at least 10 min at room temperature; separated plasma was then dispensed into polypropylene-labelled tubes and stored at −80°C until the bioanalysis was carried out.

##### Chemicals and reagents

Pure daptomycin and [^2^H_5_]-daptomycin trifluoroacetic acid salt were purchased from ALSA CHIM (Illkirch Graffenstaden, France). Water for HPLC-PLUS, methanol and formic acid was from Carlo Erba (Milano, Italy); isopropanol was from Merck (Milano, Italy). All chemicals were of analytical grade.

Drug-free human plasma used for the preparation of calibrators and control samples were obtained from the Department of Transfusion Medicine, Fondazione IRCCS Policlinico San Matteo, Pavia.

##### Calibration standards and quality control preparation

Six calibrators (A, B, C, D, E and F) were prepared in drug-free human plasma by serial dilutions: the most concentrated calibrator A (100 µg/mL) was obtained by adding 100 µL of 5 mg/mL daptomycin in methanol (first stock solution) to 4900 µL of plasma in a 10-mL Eppendorf tube^®^. The other calibrators (from 50 to 3.12 µg/mL) were prepared by mixing 300 µL of plasma with 300 µL of the previous sample, in 1.5- mL Eppendorf tubes. A second daptomycin stock solution (5 mg/mL) was used to obtain the quality controls (Qcs) at three different levels: QcH (80 µg/mL), QcM (40 µg/mL) and QcL (4 µg/mL), by serial dilution in plasma. The first one (QcH) was prepared by diluting 80 µL of the second daptomycin stock solution (5 mg/mL) with 4920 µL of plasma in a 10-mL Eppendorf tube.

##### Sample preparation

Just before the analysis, patient plasma samples were thawed, and calibrators and quality controls were prepared fresh in the same matrix. Each sample was processed by protein precipitation: 50 µL of plasma was prepared in pre-labelled Eppendorf tubes^®^. Subsequently, 300 µL of a solution containing the internal standard (IS: daptomycin-d5, 2.5 µg/mL in methanol: isopropanol = 90:10, 0.1% HCOOH) was added to promote sample clean-up, followed by vortexing (1 min) and centrifugation (10,400 rpm—10 min). In total, 200 µL of the supernatant was transferred to HPLC vials, and 5 µL was injected into the chromatographic column.

##### HPLC-MS/MS assay

The HPLC system used was a Thermo Scientific quaternary pump^®^ (Accela pump; Thermo Fisher Scientific, San Francisco, CA, USA) interfaced with an autosampler (Accela autosampler). The analytical column was a Zorbax SB C18 (4.6 × 75 mm; 3.5 µm), heated at 55°C. The mobile phases consisted of an acidified methanol/isopropanol solution (90/10 = vol/vol, HCOOCH 0.1%: mobile phase A) and acidified H_2_O (0.1% HCOOH, mobile phase B), eluted in gradient mode; the flow rate was 700 µL/min. From 0 to 0.5 min, the mobile phases were 30:70 (A:B), followed by 100% B from 1.00 to 3.0 min. From 3.10 to 4.50 min, the column was again equilibrated to the initial conditions.

Mass analysis was performed using a TSQ Quantum Access mass spectrometer system (Thermo Scientific, San Francisco, CA, USA) equipped with an electrospray interface and operated in the positive ionisation mode, following the transition *m*/*z* 811.21 >>640.82; 159.19 and *m*/*z* 813 >>641.3 for daptomycin and IS, respectively.

Xcalibur 2.07 and Lcquan2.5.6 software from Thermo Scientific (San Jose, CA, USA) were utilized for the LC-MS/MS system control, data acquisition and data analysis. Calibration curves were generated using weighted (1/*x*^2^) linear regression curves. Daptomycin was identified with a combination of retention times and specific Multiple Reaction Monitoring (MRM) transitions; the corresponding amounts were quantitated by normalizing the peak area to the IS, and concentration was calculated from the respective calibration curves.

##### Pharmacokinetics analysis

Daptomycin plasma concentration–time data were analysed using a conventional two-compartment model and first-order elimination with the software WinNonlin (version 8.0; Pharsight Corp., Mountain View, CA, USA). The elimination half-life (*t*_1/2_) was calculated as 0.693/kel, and the constant kel (the rate constant associated with the terminal elimination phase) was determined from a regression analysis of the semi-logarithmic daptomycin plasma concentration versus time data obtained 4–24 h (three data points) after infusion initiation. Daptomycin trough concentrations (*C*_trough_), maximum concentrations (*C*_max_) and volume of distribution at steady state (*V*_d,ss_, mL/kg) were also calculated. AUC_0–24_ determined on the fifth day of treatment, when the steady-state conditions were achieved, were calculated and compared between the two different groups of patients. Daptomycin clearance was estimated as the ratio between the daily dose and the AUC_0–24_; a correlation between creatinine clearance (by use of the Cockcroft-Gault equation) and daptomycin clearance was analysed.

The pharmacokinetic/pharmacodynamic (PK/PD) parameter evaluated was AUC/MIC (both >666 and >1061), percentage of patients attaining the threshold value was reported. The occurrence of CK elevation above the normal value (>170 mU/mL) and eosinophilic pneumonia was evaluated.^17^

## Results

Twenty-two patients and twenty-three infectious endocarditis episodes were included in this study (Table 1). Native valve IE was diagnosed in 9 patients, prosthetic valve IE was diagnosed in 10 patients, 3 patients had concomitant intracardiac device infections (2 ICD/1 PM). Eight out of these ten patients with prosthetic infections received 11–12 mg/kg daptomycin daily.

**Table 1.** Characteristics of patients with infective endocarditis.

Variables	All	Group A 8–10 mg/kg	Group B 11–12 mg/kg
Age (years)	68.5 (1–3 IQR 56.75–74)	69 (1–3 IQR 57.5–80)	64 (1–3 IQR 56–71.25)
Males *n* (%)	16 (69)	11 (73)	10 (71)
Weight	70 (1–3 IQR 58.5–79.75)	70 (1–3 IQR 58.5–80)	72.5 (1–3 IQR 62.5–83.5)
Creatinine	0.96 (1–3 IQR 0.87–1.16)	0.9 (1–3 IQR 0.86–1.06)	1,06 (1–3 IQR 0.90–1.22)
Native valve *n* (%)	9 (39.2)	4 (26.7)	6 (42.9)
-aortic valve	5 (55.5)	1 (25)	4 (66.7)
-mitral valve	4 (44.5)	3 (75)	2 (33.3)
Prosthetic valve *n* (%)	11 (47.8)	9 (60)	7 (50)
-aortic valve	7 (63.6)	7 (77.8)	4 (57.1)
-mitral valve	4 (36.4)	2 (22.2)	3 (42.9)
CIED-related infective endocarditis	3 (13%)	2 (13.3%)	1 (7.1%)
Microbial identification			
-*Staphylococcus aureus*	13 (56.5%)	8 (53.3%)	7 (50%)
- MSSA	7 (53.8%)	6 (75%)	3 (42.9%)
- MRSA	6 (46.2%)	2 (25%)	4 (57.1%)
*- Staphylococcus epidermidis*	7 (30.4%)	5 (33.3%)	5 (35.8%)
- MSSE	2 (28.6%)	1 (20%)	2 (40%)
- MRSE	5 (71.4%)	4 (80%)	3 (60%)
- *Staphylococcus capitis*	1 (4.3%)	1 (6.7%)	1 (7.1%)
- *Staphylococcus lugdunensis*	1 (4.3%)	0	1 (7.1%)
- *Staphylococcus haemolyticus*	1 (4.3%)	1 (6.7%)	0

CIED, cardiac implantable electronic device; IQR, interquartile rate; MRSA, methicillin resistant Staphylococcus aureus; MRSE, methicillin-resistant Staphylococcus epidermidis; MSSA, methicillin susceptible Staphylococcus aureus; MSSE, methicillin susceptible Staphylococcus epidermidis.

Bacteria isolated trough blood cultures included *S. aureus* (13), of which 6 (46.2%) were methicillin-resistant (MR); *S. epidermidis* (7), of which 5 (71.4%) were MR, *S. haemolyticus* MR (1 case), *S. capitis* MR (1 case) and *S. lugdunensis* methicillin sensible (MS) (1 case). MIC values were <0.5 µg/mL in all cases. The length of treatment with daptomycin was 28 days (1–3 IQR 28–42).

Ten patients underwent surgical treatment, and nine could not undergo surgery despite indications deemed as high risk (three patients with a Bentall prosthesis, four patients with prosthetic valve, one patient with native valve endocarditis and one patient with ICD).

All patients displayed a microbiologic response with negative blood cultures by day 5 in median (1–3 IQR 3–8). All patients were alive at 30 days, no recurrences were observed in patients with native valve endocarditis at 30 days and/or in those surgically treated. Three patients with an infection of the Bentall prosthesis, and one patient with an ICD that could not be extracted, continued chronic suppressive therapy after hospital discharge with oral antimicrobial treatment. Among the 4 patients with prosthetic valve disease that could not be operated, 2 showed a long-term response to antimicrobial treatment (8 weeks intravenous and oral subsequently), while one patient had a recurrence of *S. capitis* infection months after the first episode (after discontinuing oral therapy) and one had two different episodes of endocarditis caused by two different microorganisms (*S. haemolyticus* and *S. epidermidis*, both MR). Adverse reactions to daptomycin were not observed throughout the study period; none of the patients presented a CK increase nor eosinophilic pneumonia during treatment.

### Pharmacokinetics

Globally, 15 patients were included in group A and 14 in group B (Table 2). Six patients received two different doses of daptomycin (as per the initial study design).

**Table 2.** Pharmacokinetics of daptomycin (data expressed as median (IQR)).

Variables	Group A (8–10 mg/kg)	Group B (11–12 mg/kg)
Daptomycin *C*_trough_ (µg/mL)	30.9 (25.6–40.1)	31.6 (27.5–46.2)
Daptomycin *C*_max_ (µg/mL)	109.1 (89.8–123)	122.2 (105.2–149.7)
Daptomycin AUC_0–24_ (µg*h /mL)	1298 (1069–1484)	1459 (1218–1711)
Daptomycin CL (L/h)	0.49 (0.37–0.57)	0.57 (0.40–0.71)
Daptomycin *K*_el_ (h^−1^)	0.041 (0.033–0.048)	0.046 (0.038–0.051)
Daptomycin *t*_1/2_ (h)	16.9 (14.2–21)	15.1 (13.6–18.2)
Daptomycin *V*_d,ss_ (L/kg)	0.16 (0.14–0.20)	0.16 (0.14–0.20)

AUC, area under the curve.

The median daptomycin *C*_trough_ was 30.9 µg/mL (IQR 25.6–40.1) and 31.6 µg/mL (IQR 27.5–46.2), and the median *C*_max_ was 109.1 µg/mL (IQR 89.8–123) and 122.2 µg/mL (IQR 105.2–149.7), respectively, for the two different dose regimens. *C*_trough_ was above 24 µg/mL in all patients except two (both in group A).

The median calculated AUC_0-24_ was 1298 (IQR 1069–1484) and 1459 (IQR 1218–1711) µg*h/mL, with the corresponding clearances of 0.49 (IQR 0.37–0.57) and 0.57 (IQR 0.40–0.71) L/h. The median *K*_el_ (h^−1^) was 0.041 (IQR 0.033–0.048) and 0.046 (IQR 0.038–0.051) h^−1^, and the median elimination half-life (*t*_1/2_) were 16.9 (14.4–21) and 15.1 (13.6–18.2) h, whereas the *V*_d,ss_ was 0.16 (L/kg) (IQR 0.14–0.20) for both the dose regimens. There was a moderate correlation between CL_DAP_ and both CL_cr_ and age; the Pearson correlation coefficient (*r*) was 0.59 and 0.35, respectively.

Among the considered pharmacokinetics parameters, AUC_0–24_ was significantly associated with dose group (beta coefficient 274, 95% CI 64–483, *p* = 0.010), age (beta coefficient 19 95% CI 9–29, *p* < 0.001) and creatinine (beta coefficient 390 95% CI 5–774 *p* = 0.047) while gender did not affect the results. Patients in dose group B have higher AUC regardless of other included covariates.

A value of AUC/MIC > 666 was reached by all patients; however, four out of 15 patients in group A and 1 out of 14 patients in group B did not reach the PK/PD target of 1061; therefore, AUC/MIC equal to or above 1061 was reached by 73.3% in group A and 92.9% in group B. Patients who did not reach the PK/PD target were two women and two men in group A and a male patient in group B, their age was variable (between 32 and 63 years old).

## Discussion

Therapeutic failures with the emergence of resistance have been reported with daptomycin use, particularly in cases of deep-seated infections, persistent bacteraemia and low-dose regimens in infectious endocarditis.^6,18–20^ To overcome this limit, higher than 6- mg/kg daptomycin doses have been endorsed although in published studies ‘high-dose’ daptomycin treatment can refer to very different daily doses, ranging from 6.1 to >12 mg/kg.^7,8,21^ Different observational studies have proved the effectiveness of higher daptomycin doses (>8 mg/kg) compared with 6 mg/kg, the former was in fact associated with higher odds of treatment success.^22,23^ In an observational study named EU-CORE that enrolled roughly 6000 patients, those with a high inoculum infection were more likely to be treated with high-dose daptomycin. A higher clinical success rate was also observed with increasing the dose in endocarditis associated with foreign body/prosthetic infection.^22^ Rosso et al. evidenced that the use of daptomycin standard dose was associated with increased 30-day mortality.^23^

Although there is agreement on the use of higher daptomycin doses, pharmacokinetic data on patients treated with different ‘higher doses’ are lacking; the present study evaluated the pharmacokinetics relative to two different higher doses, namely 8–10 versus 11–12 mg/kg daily. The present study confirms linear daptomycin pharmacokinetics up to doses of 12 mg/kg when administered once daily, as it has previously been reported in healthy volunteers.^24^ Also, at higher doses, daptomycin demonstrates a limited volume of distribution (*V*_d,ss_: 0.16 L/kg), according to the estimated 0.1–0. 2 L/kg of body weight.^8^

In patients with *Staphylococcus* spp. endocarditis and normal kidney function, all patients achieved the target of AUC/MIC < 666, conversely a daptomycin daily dose corresponding to 8–10 mg/kg attained the target PK/PD value of 1061 in 75% of patients in the 8 to 10-mg/kg group and in more than 90% of those in the 11 to 12-mg/kg group, with *Staphylococcus* strains MIC of 0.5 µg/mL. Considering that the daptomycin MIC breakpoint of 1 mg/L for *Staphylococcus* spp.,^25^ the PK/PD target would be reached in a lower percentage of cases, where the MIC is higher than 0.5 µg/mL.

In the clinical setting though, the picture is more complex. Bhavnani and co-authors have shown that there is not a clear-cut AUC/MIC value that directly correlates with clinical response, as other factors such as concomitant kidney diseases, low albumin level and severity of infection can interfere with the clinical response.^17^ In accordance, an increase in daptomycin MIC has been observed in *Staphylococcus aureus* strains isolated in biofilm, compared with a strain isolated in blood cultures, as a result of this the concentration of antimicrobial needed to overcome the infection within the biofilm may not be adequate.^11^ In a biofilm, daptomycin concentration has been documented to penetrate 10% of the biofilm structure.^26^ Moreover, concentrations required for bactericidal activity may be increased in the case of bacteria in their stationary phase.^27^

For these reasons, as 25% of the patients in the 8- to 10-mg/kg group did not reach the PK/PD target, it could be reasonable to use doses up to 12 mg/kg daily for the treatment, in particular in case of prosthetic infections in which biofilm productions can be predicted or in high-inoculum infections (i.e., cardiac abscess). All patients here described responded to the treatment, without evidence of breakthrough infections while on treatment although patients with prosthetic infections that could not have the device removed usually were maintained in a suppressive regimen after discharge.

At multivariable analysis, age was independently associated with daptomycin AUC_0–24_. The systemic exposure to daptomycin decreases with decreasing age, indicating that the clearance of the drug is faster in younger individuals, Dvorchik and Damphousse observed that the systemic exposure to daptomycin was 58% higher in elderly individuals (>75 years) when compared with younger patients (18–30 years).^28^ This indication can be in support of treatment of younger patients when higher doses could be indicated. Also, clearance was a covariate independently associated with AUC_0–24_. Therefore, as CL_cr_ correlated with CL_DAP_, those conditions such as severe infection that can be associated with hyperdynamic states and altered Cl_cr_ may lead to altered disposition of daptomycin.^29^

Several studies reported an association between plasma concentration of daptomycin and efficacy or adverse effects, in particular creatinine phosphokinase elevation and eosinophilic pneumonia.^17,30,31^ In particular, patients whose trough concentrations of daptomycin were more than 24.3 mg/mL showed a higher frequency of creatinine phosphokinase elevation, indicating the occurrence of rhabdomyolysis that is one of the typical adverse effects induced by daptomycin administration.^17^ No adverse reactions due to daptomycin were reported in this study despite the fact that all patients except for two, displayed a *C*_trough_ below 24 µg/mL. Given the possibility of adverse effects at higher daptomycin doses, the therapeutic threshold (that is AUC/MIC >666 or >1061) should be weighed in the context of the mortality and severe morbidity associated with these types of serious infections.

This study had a few limitations. The monocentric study design and the limited sample size should be acknowledged. Moreover, patients with coagulase-negative *Staphylococcus* species have been included besides *S. aureus*. Although clinical^32^ and experimental data support the use of daptomycin for *S. epidermidis*^33^ and daptomycin MIC values are comparable for both species,^11^ the study could not evaluate the efficacy for each pathogen, which may have affected the results.

## Conclusion

In conclusion, the study evidences that patients were successfully treated in both groups. From PK/PD point of view, all patients reached the value of AUC/MIC > 666, while roughly 70% of patients in group A and 90% in group B reached the target value of AUC/MIC>1061. Even if this cut-off value is arbitrary, the 11- to12-mg/kg daily dose could be taken into consideration in case of serious infections characterised by high-inoculum infections or in case of prosthetic valve infections.

## Declarations

**Ethics approval and consent to participate:** The study was approved by Comitato Etico Pavia (P-20190113104). Each participant signed a written informed consent to participate in this study.

**Consent for publication:** Not applicable.

**Availability of data and materials:** Data will be available upon request to corresponding author.
